# Vancomycin-resistant enterococci and coagulase-negative staphylococci prevalence among patients attending at Felege Hiwot Comprehensive Specialized Hospital, Bahir Dar, Ethiopia

**DOI:** 10.1371/journal.pone.0249823

**Published:** 2021-04-08

**Authors:** Degu Ashagrie, Chalachew Genet, Bayeh Abera

**Affiliations:** 1 Diagnostic Medical Laboratory Unit, Felege-Hiwot Comprehensive Specialized Hospital, Bahir Dar, Ethiopia; 2 Department of Medical Laboratory Science, College of Medicine and Health Science, Bahir Dar University, Bahir Dar, Ethiopia; Nitte University, INDIA

## Abstract

**Background:**

Vancomycin resistant enterococci (VRE) and vancomycin resistance coagulase negative staphylococci (VRCoNS) are common pathogens causing difficult to treat health care associated infections (HAI). Hence, the World Health Organization listed VRE as one of the high priority pathogens for new antibiotic discovery and antimicrobial resistance surveillance. Despite this, data on the prevalence of VRE and VRCoNS in Ethiopia is scarce. Thus, the present study determined prevalence of VRE and VRCoNS among patients attending Felege-Hiwot comprehensive specialized hospital, Ethiopia.

**Methods:**

A hospital based cross-sectional study was conducted on 384 patients selected conveniently from February to March 2020. Data on demographic and clinical variables were collected using a structured questionnaire by face-to-face interview. Simultaneously urine, venous blood and wound swab were collected and processed following standard bacteriological technique. Antimicrobial susceptibility test was performed by minimum inhibitory concentration method using E-test for vancomycin and Kirby-Bauer disc diffusion method for other classes of antibiotics. Data was entered and analyzed using SPSS version 23. Logistic regression was performed to identify factors associated with VRE infection. P. value < 0.05 was considered as statistically significant.

**Results:**

The prevalence of enterococci and CoNS were 6.8% and 12% respectively. The prevalence of VRE was 34.61% (9/26), while all CoNS (46 isolates) were susceptible to vancomycin. The majority (66.7%) of VRE was isolated from blood samples. Furthermore all VRE (100%), 58.8% of vancomycin susceptible enterococci and 45.7% of CoNS were multidrug resistant (MDR). Having educational level of secondary school and below (AOR = 12.80, CI = 1.149–142.5), previous exposure to catheterization (AOR = 56.0, CI = 4.331–724.0) and previous antibiotic use practice (AOR = 26.25, CI = 3.041–226.2) were a significant associated explanatory factor for VRE infection.

**Conclusions:**

The prevalence of vancomycin resistance enterococci, which is also multidrug resistant, was significantly high. Though no vancomycin resistance CoNS detected, the MDR level of CoNS was high. Thus to limit enterococci and CoNS infections and MDR development, focused infection prevention measures should be implemented.

## Background

Antimicrobial resistance (AMR), which is a complex global public health challenge, reduces the efficacy of antibiotics making bacterial infection treatment difficult, costly, or even impossible [1–3]. Vancomycin, a glycopeptide antibiotic discovered around 1950s, has been used as the last resort antibiotic for the treatment of multidrug-resistant Gram positive bacterial infections including those caused by enterococci and coagulase-negative staphylococci (CoNS) [1, 4–6]. Depending on infection type and diagnostic methods used, enterococci and CoNS are responsible for 0.6%-10.7% [7–12] and 7.8% to 42.8% [10–16] of bacterial infections respectively.

Resistance development by enterococci and CoNS for different antibiotics including vancomycin is a growing challenge globally [3, 17–19]. Vancomycin resistance for CoNS and enterococci were reported immediately after vancomycin was discovered [4, 5, 20, 21]. But now, different studies reported the wide spread of vancomycin resistance coagulase negative staphylococci (VRCoNS) and vancomycin resistant enterococci (VRE) globally [18, 19, 22]. Moreover VRE was listed as a high priority pathogen by the World Health Organization (WHO) in 2017 for new antibiotic discovery, development and AMR surveillance [23].

Despite limited virulence factors associated with VRE and VRCoNS, they are among the five common health care associated infections (HAI) causative agents mainly in patients with indwelling/implanted medical device, old age and/or immunocompromised [17, 20, 24, 25]. With varying degree of involvement, VRE and VRCoNS can cause different infections such as urinary tract infection (UTI), wound infection, blood stream infection (BSI), endocarditis, neonatal sepsis, meningitis, ocular infection and otitis media [4, 5, 17, 26, 27]. Such infections are causing substantial clinical burden with increasing trend globally [2, 4, 6, 26, 28]. Furthermore vancomycin resistant strains increase mortality, hospital stay, infection recurrence and treatment costs than sensitive strains [29–34].

The prevalence of VRE (9.8%-26.4%) [26, 33, 35–38] and VRCoNS (0%-4.4%) [15, 39–42] is different globally and showed an increasing trends overtime [26, 31]. Particularly the impact of VRE and VRCoNS infections are more prominent in developing countries, including Ethiopia, where there is less organized control over the use of broad spectrum antibiotics allowing the development of vancomycin resistance [18, 19, 28]. A meta-analysis on VRE [19] and VRCoNS [18] in Ethiopia reported 14.8% and 11% resistance to vancomycin respectively. Likewise different prevalence studies among different study populations and clinical specimens in Ethiopia reported 6.7%-22.7% VRE [7, 8, 43, 44] and 0%-15.4% [10, 16, 45] VRCoNS. Besides presence of underlying disease condition, prolonged hospitalization, previous exposure to catheterization, old age, history of hospital admission and previous antibiotic treatment increased the risk of acquiring VRE and VRCoNS [8, 13, 33, 43].

To reduce the impact, emergency and spread of VRE and VRCoNS, surveillance and local prevalence studies are recommended by WHO [1, 23] and other previous studies [7, 13, 37, 41, 42] because it allows to know the actual extent of the problem for designing evidence based intervention. Studies are limited on the prevailing prevalence of VRE and VRCoNS in the study area. Thus the present study was intended to determine the prevalence of VRE, VRCoNS and associated factors among patients suspected to have UTI, septicemia and wound infection at Felege-Hiwot comprehensive specialized hospital (FHSCH), Ethiopia.

## Methods

### Study design and period

A hospital based cross sectional study was conducted at FHCSH, Bahir Dar, North West Ethiopia from February to March 2020. FHCSH, established in 1952, serves for more than 5 million people living in Bahir Dar city and surrounding zones. The hospital has 13 wards, 430 beds and about 531 health professionals.

The source population was all patients attending at in-patient and outpatient departments of FHCSH and clinically diagnosed as having UTI, septicemia or wound infection. Meanwhile the study population was patient clinically diagnosed as having UTI, septicemia or wound infection at FHCSH in the study period.

### Inclusion and exclusion criteria

All patients clinically diagnosed as having UTI, septicemia or wound infection who gave written informed consent or assent was included in the study. But patients who were on antibiotic treatment at the time of data collection were excluded from the study.

### Sample size and sampling technique

A total of 384 study participants were included using a single population proportion formula. Since there was no similar study done on prevalence of VRE and VRCoNS, 50% prevalence was used to calculate the sample size at 5% margin of error and 95% level of confidence. A convenient sampling technique was used to enroll study participants. Any patients who fulfilled the inclusion criteria were included consequently until the required number was achieved

### Demographic and clinical data collection

Demographic data such as sex, age, marital status, residence and clinical data including types of infection, history of hospital admission and Catheterization were collected using a pre-tested structured questionnaire developed for this research by face-to-face interview from patients clinically diagnosed as having UTI, septicemia or wound infection by attending physician from inpatient and outpatient department of the hospital.

### Clinical specimen collection

After the interview, urine, venous blood or wound swab were collected aseptically from patients clinically diagnosed as having UTI, septicemia or wound infection respectively. From non-catheterized patients, 5–10 ml of clean-catch midstream urine specimen was collected by sterile urine cup. From catheterized patients, the same volume of urine was aseptically collected via a urethral or supra pubic catheter line and port to sterile urine cup. Ten ml, 5ml and 1ml of venous blood was collected from adults, children and infants respectively using culture bottles with Tryptone soya broth (Oxoid; Hampshire UK). Moreover the wound swab was collected using a sterile cotton swab. All collected specimens were transported to Bahir Dar University Medical Microbiology research laboratory. All wound swab specimen was transported using Amie’s transport medium [46].

### Bacterial isolation and identification

Urine specimen was processed within two hours of collection. A 0.001ml urine specimen was inoculated into blood agar plate using calibrated wire loop and incubated overnight at 37˚C. Plates with 10^5^ CFU/mL for non-catheterized and 10^3^−10^5^ for catheterized urine were considered as significant bacteriuria and further sub cultured into mannitol salt agar plate and blood agar plate to isolate *Enterococcus* spp. and CoNS. The blood culture bottle incubated at 37°C was observed daily for 7 consecutive days for microbial growth evidenced by presence of hemolysis, gas formation or media color change. But if there is no visible growth within 7 days, Gram stain was performed and if there was no Gram positive bacteria detected, it was considered as negative. Culture bottles which showed growth were opened aseptically and small amount of broth was taken using sterile wire loop and sub cultured into Blood agar plate and mannitol salt agar plat. The wound swab was inoculated into blood agar plate and mannitol salt agar plate and incubated at 37°C for 24 hours. All culture media used were Oxoid; Hampshire UK. Subsequent identification of *Enterococcus* spp. was done based on Gram stain, hemolysis, esculin hydrolysis (in bile-esculin agar), catalase test, L-pyrrolidonyl-β-arylamidase (PYR) test and salt tolerance test. Similarly CoNS identification was done based on colony characteristics, Gram stain, catalase and coagulase tests following the WHO basic laboratory procedures in clinical bacteriology [46]

### Antibiotic susceptibility testing

Based on Clinical Laboratory Standards Institute (CLSI) guideline, vancomycin susceptibility testing for both enterococci and CoNS isolates was performed by minimum inhibitory concentration (MIC) method on Muller-Hinton agar (Oxoid; Hampshire UK) using E-test (bioMérieux SA, Marcy-1’Étoile, France) after adjusting the test inoculum using a 0.5 McFarland standard [47]. Susceptibility results of vancomycin was interpreted as sensitive, intermediate and resistant [47]. Antimicrobial susceptibility test (AST) for antibiotics other than vancomycin were done using Kirby-Bauer disk diffusion method on Muller-Hinton agar (Oxoid; Hampshire UK) by adjusting the turbidity with 0.5 McFarland standard based on 2019 CLSI guideline. Enterococci and CoNS isolates were tested against ampicillin (10μg), ciprofloxacin (5μg), chloramphenicol (30μg), erythromycin (15μg) and tetracycline (30μg). Antibiotics used were selected based on CLSI guideline [47], local prescription pattern, availability and all were Oxoid (Basingstoke, Hampshire, England). Results of AST were interpreted following CLSI guideline [47].

### Quality control

Before data collection, the questionnaire was pre-tested and every questionnaire was cheeked for its completeness after collection. To avoid contamination of the blood sample by skin colonizing CoNS during collection, 10% tincture iodine was used to clean the sample collection area. All culture media was prepared following the manufacturer’s instructions. A sample of culture media plate prepared from each batch was incubated at 37°C for 24 hour to cheek for sterility. Before inoculation, the culture media was visually inspected for any microbial growth or deterioration. Moreover *E*. *faecalis* ATCC 29212, *S*. *aureus* ATCC 25923 and *S*. *aureus* ATCC 29213 standard strains were used as quality control [47].

### Data analysis

Collected data were entered and analyzed using Statistical Package for Social Science 23 (IBM Corp Released 2011.IBM SPSS statistics. Armonk, NY: IBM Corp). Descriptive statistics like frequency and percentage were computed to determine the magnitude of VRE, VRCoNS and demographic variables. Multivariable analysis was done to determine factors associated with VRE infection and P-value <0.05 was considered as statistically significant.

### Ethics considerations

Ethical clearance was obtained from the Institutional Review Board (IRB) of Bahir Dar University, College of Medicine and Health Sciences protocol number 0012/2020. Moreover before data collection, informed written consent was obtained from each study participant with the age of 16 years and above. From the study participants having age less than 16 years, a written assent was obtained from their parents or guardians. In addition, all the information obtained from the study subjects was registered by code to maintain confidentiality and significant culture results were communicated with responsible physician

## Results

### Demographic characteristics of the study participants

From 384 study participants, 199 (51.8%) were females. The age of study participants ranges from 1–83 years with a mean age of 23 years old. Among respondents, 65.1% were urban residents. Seventy-two (18.8%) study participants were culture positive either for enterococci or CoNS. Moreover, there was no double infection by enterococci and CoNS in a single patient (Table 1).

**Table 1:** Demographic characteristics of study participants (n = 384) and bacterial isolation rate at FHCSH, Bahir Dar, Ethiopia, 2020.

Variables	Positive: N (%)	Negative: N (%)	Frequency (%)
Sex			
Male	32 (17.2)	154 (82.8)	186 (48.4)
Female	40 (20.2)	158 (79.8)	198 (51.6)
Age (in years)			
0–15	28 (17.4)	133 (82.6)	161 (41.9)
16–30	14 (15.2)	78 (84.8)	92 (23.9)
31–45	14 (21.9)	50 (78.1)	64 (16.7)
46–60	10 (34.5)	16 (61.5)	26 (6.8)
≥ 61	6 (14.6)	35 (85.4)	41 (10.7)
Educational status			
Can’t read and write	3 (13.0)	20 (87.0)	23 (6.0)
Primary	25 (21.2)	93 (78.8)	118 (30.7)
Secondary	10 (14.3)	60 (85.7)	70 (18.2)
College	9 (20.5)	35 (79.5)	44 (11.5)
Under school age	25 (19.4)	104 (80.6)	129 (33.6)
Marital status			
Married	20 (17.9)	92 (82.1)	112 (29.2)
Unmarried	47 (19.3)	196 (80.7)	243 (63.3)
Divorced/Widowed	5 (17.2)	24 (82.8)	29 (7.5)
Residence			
Rural	21 (15.7)	113 (84.3)	134 (34.9)
Urban	51 (20.4)	199 (79.6)	250 (65.1)
Occupation			
Employed	6 (19.4)	25 (80.6)	31 (8.1)
Merchant	17 (26.2)	48 (73.8)	65 (16.9)
Dial laborer	8 (28.6)	20 (71.4)	28 (7.3)
Farmer	6 (15.0)	34 (85.0)	40 (10.4)
Under age	35 (15.9)	185 (84.1)	220 (57.3)
**Total**	**72 (18.8)**	**312 (81.2)**	**384 (100)**

### Proportion of vancomycin resistance enterococci and coagulase negative staphylococci

The prevalence of *Enterococcus* spp. and coagulase negative *Staphylococcus* spp. infections were 6.8% (26 isolates) and 12% (46 isolates) respectively. The proportion of VRE was 34.6% (9/26). On the other hand, all CoNS isolates (100%) were susceptible to vancomycin. Out of 26 total enterococci isolates, 76.9% was isolated from urban residents. Likewise among 46 total CoNS isolates, 33 (71.7%) were isolated from unmarried study participants. Among three clinical specimens, the highest isolation rate of enterococci and CoNS were from urine and blood with 9.5% and 13.2% respectively (Table 2).

**Table 2:** Prevalence of *Enterococcus* spp. and coagulase negative *Staphylococcus* spp. in relation to different variables among patients attending at FHSCH, Bahir Dar, Ethiopia, 2020.

Variables	Enterococci infection: N (%)	CoNS infection: N (%)	VRE proportion: N (%)
Sex			
Male (n = 186)	10 (5.4)	22 (11.8)	3 (30)
Female (n = 198)	16 (8.1)	24 (12.1)	6 (37.5)
Age (in years)			
0–15 (n = 161)	4 (2.5)	24 (14.9)	0 (0)
16–30 (n = 92)	6 (6.5)	8 (8.7)	3 (50)
31–45 (n = 64)	7 (10.9)	7 (10.9)	3 (42.9)
46–60 (n = 26)	5 (19.2)	5 (19.2)	1 (20)
≥ 61 (n = 41)	4 (9.8)	2 (4.9)	2 (50)
Educational status			
Can’t read and write (n = 23)	2 (8.7)	1 (4.3)	1 (50)
Primary (n = 118)	11 (9.3)	14 (11.7)	3 (27.3)
Secondary (n = 70)	3 (4.3)	7 (10)	1 (33.3)
College (n = 44)	5 (11.4)	4 (9.1)	4 (80)
Under school age (n = 129)	5 (3.9)	20 (15.5)	0 (0)
Marital status			
Married (n = 112)	8 (7.1)	12 (10.7)	3 (37.5)
Unmarried (n = 243)	14 (5.8)	33 (13.6)	4 (28.6)
Divorced/Windowed (n = 29)	4 (13.8)	1 (3.5)	2 (50)
Residence			
Rural (n = 134)	6 (4.5)	15 (11.2)	2 (33.3)
Urban (n = 250)	20 (8)	31 (12.4)	7 (35)
Occupation			
Employed (n = 31)	3 (9.7)	3 (9.7)	2 (66.7)
Merchant (n = 65)	6 (9.2)	11 (16.9)	3 (50)
Dial laborer (n = 28)	4 (14.3)	4 (14.3)	1 (25)
Farmer (n = 40)	3 (7.5)	3 (7.5)	1 (33.3)
Under age (n = 220)	10 (4.6)	25 (11.4)	2 (20)
Clinical specimen used			
Urine (n = 126)	12 (9.5)	14 (11.1)	2 (16.7)
Blood (n = 227)	12 (5.3)	30 (13.2)	6 (50)
Pus (n = 31)	2 (6.5)	2 (6.5)	1 (50)
Total	**26 (6.8%)**	**46 (12%)**	**9 (34.6)**

**Key:** CoNS = Coagulase negative staphylococci

### Antimicrobial resistance profile of enterococci and CoNS

The antimicrobial resistance profile of enterococci and CoNS to other classes of antimicrobials are illustrated in Table 3. Both enterococci and CoNS revealed the highest rate of resistance for ampicillin with 96.2% and 76.1% respectively. On the other hand medium resistance level was observed in enterococci and CoNS for ciprofloxacin (53.9%) and chloramphenicol (43.5%) respectively (Table 3).

**Table 3:** Antimicrobial resistance profile of *Enterococcus* spp. and coagulase negative *Staphylococcus* spp. isolated among patients attending at FHSCH, Bahir Dar, Ethiopia, 2020.

Antibiotic disc	Resistance isolates number (%)	
	*Enterococcus* Spp.: N (%)	CoNS: N (%)
Ampicillin	25 (96.2)	35 (76.1)
Chloramphenicol	16 (61.5)	20 (43.5)
Ciprofloxacin	14 (53.9)	21 (45.7)
Erythromycin	17 (65.4)	23 (50.0)
Tetracycline	15 (57.7)	25 (54.4)

Multi drug resistance (MDR) bacteria in this study refer to resistance for ≥ 3 antibiotics in different classes. Hereby, the overall multidrug resistance (MDR) level of enterococci was 73.1%. Among enterococci isolates, all VRE and 58.8% vancomycin susceptible enterococci (VSE) showed MDR. Regarding CoNS, 21 (45.7%) isolates were MDR (Table 4).

**Table 4:** Multidrug resistance level of VRE, VSE and CoNS isolates among patients attending at FHSCH, Bahir Dar, Ethiopia, 2020.

Bacterial isolate	Level of antibiotic resistance			
	R3: N (%)	R4: N (%)	R5: N (%)	MDR: N (%)
VRE (n = 9)	0 (0)	6 (66.7)	3 (33.3)	9 (100)
VSE (n = 17)	1 (5.9)	7 (41.2)	2 (11.8)	10 (58.8)
CoNS (n = 46)	13 (28.3)	8 (17.4)	0 (0)	21 (45.7)

**Key:** VRE: Vancomycin Resistance Enterococci; VSE: Vancomycin Susceptible Enterococci, CoNS: Coagulase Negative Staphylococci; **R3** = Resistance to three antibiotics; **R4** = Resistance to four antibiotics, **R5** = Resistance to five antibiotics, MDR = resistance for ≥ 3 antibiotics in different class

### Multivariable analysis on risk factors of VRE infection

On multivariable analysis, educational level, previous exposure to catheterization and previous antibiotic use practice were significant associated explanatory factors for VRE infection. Patients with previous history of catheterization and antibiotic use had 56 and 26 times more chance to have VRE infection than their counterparts. Though, it was not significant, higher VRE infection was found in females compared to male patients (Table 5).

**Table 5:** Multivariable analysis on the associated factors of VRE infection among patients attending at FHSCH, Bahir Dar, Ethiopia, 2020.

Variables	Prevalence of enterococci		Total	AOR (95% CI)	P-Value
	VRE: n (%)	VSE: n (%)			
Sex					
Male	3 (30)	7 (70)	10	1	
Female	6 (37.5)	10 (62.5)	16	1.815 (0.340–9.687)	0.486
Age (years)					
≤ 30	3 (30)	7 (70)	10	1	
31–45	3 (42.9)	4 (51.1)	7	1.750 (0.233–13.15)	0.587
46–60	1 (20)	4 (80)	5	0.583 (0.044–7.661)	0.682
≥61	2 (50)	2 (50)	4	2.333 (0.216–25.24)	0.486
Educational level					
Secondary & below	5 (23.8)	16 (76.2)	21	12.80 (1.149–142.5)	0.038
Above secondary	4 (80)	1 (20)	5	1	
Marital status					
Married	3 (37.5)	5 (62.5)	8	1	
Unmarried	4 (28.6)	10 (71.4)	14	0.667 (0.106–4.206)	0.666
Divorced/Widowed	2 (50)	2 (50)	4	1.667 (0.147–18.87)	0.68
Residence					
Rural	2 (33.3)	4 (66.7)	6	1	0.94
Urban	7 (35)	13 (65)	20	1.077 (0.156–7.42)	
Occupation					
Employed	2 (66.7)	1 (33.3)	3	8.0 (0.459–139.29)	0.154
Merchant	3 (50)	3 (50)	6	4.0 (0.431–37.10)	0.223
Dial laborer/farmer	2 (28.6)	5 (71.4)	7	1.6 (0.168–15.27)	
Student/underage	2 (20)	8 (80)	10	1	
Infection type					
Blood stream	6 (50)	6 (50)	12	3.667 (0.666–20.19)	0.135
UTI/Wound	3(21.4)	11 (78.6)	14	1	
History of admission					
<24hrs	4 (57.1)	3 (42.9)	7	1	
>24hr	3 (60)	2 (40)	5	1.125 (0.109–11.59)	0.921
Catheterization					
Catheterized	7 (87.5)	1 (12.5)	8	56.0 (4.331–724.0)	0.002
Non-catheterized	2 (11.1)	16 (88.9)	18	1	
Antibiotic used history					
Yes	7 (77.8)	2 (22.2)	9	26.25 (3.041–226.2)	0.003
No	2 (11.8)	15 (88.2)	17	1	

**Key**: VRE: Vancomycin Resistance Enterococci; VRE: Vancomycin Susceptible Enterococci

## Discussion

Health care associated infection due to VRE and VRCoNS is a growing challenge globally. These problems mainly affect African countries including Ethiopia coupled with the presence of high AMR to commonly prescribed antibiotics [3, 48]. The prevalence of enterococci infection in the present study was comparable with Gondar, Ethiopia (6.2%) [43] but lower than the United Kingdom (14.7%) [36]. This might be due to differences in clinical sample analyzed, sample size, background characteristics of the study participants and bacterial detection method. On the other hand, the prevalence of enterococci in the present study was slightly higher than studies in Jimma, Ethiopia [7] (5.5%) and Addis Ababa, Ethiopia (3.6%) [8]. This variation might be because of an increase in enterococcal infection through time. Furthermore, differences in clinical sample analyzed, sample size, background characteristics of the study participants and bacterial detection method may account the differences. The prevalence of CoNS infection in this study was comparable with previous studies in Ethiopia [16, 45], and Pakistan [41] which reported 14.5%-14.7%, and 12.7% respectively. In contrast, higher prevalence of 31.6% was reported from a study conducted in Gondar, Ethiopia [39].

Potential local factors for acquiring VRE infection were explored. Therefore, educational level, catheterization practice and previous antibiotic use were significantly associated with VRE infection. A similar finding was reported from other parts of Ethiopia [43, 44]. Even though equal amount of clinical specimen was not taken, the highest proportion of enterococci in the present study was isolated from blood, urine, and pus. A comparable finding was reported from Gondar, Ethiopia [43]. This might reflect the role of enterococci in causing UTI, BSI and wound infection [5]. Moreover in the present study, the highest proportion of CoNS was isolated from blood (65.2%). A comparable finding was reported by previous studies in India (48.7%) and Pakistan (45.9%) [40, 41].

The prevalence of VRE in the present study was comparable with studies done in Gondar, Ethiopia (41.7%) [43]. However it was significantly higher than previous studies done in other parts of Ethiopia which reported 6.7% to 22.7% [7, 8, 44]. Similarly, it was higher than studies done in different parts of the world including South Korea, Iran, United Kingdom, Nepal and Turkey which reported 9.8%- 26.4% [33, 35–38]. This high VRE finding in the present study might indicate an increasing trend in VRE which is supported by studies reporting steady increase in VRE through time [26, 49]. On the other hand, vancomycin resistant CoNS was not detected in the present study. This finding was coherent with previous studies in Ethiopia [16], India [40], China [42] and Tunisia [50] which all reported no VRCoNS isolates. However, it was reported that VRCoNS was detected in studies conducted in Ethiopia [10, 45], Pakistan [41], and Iran [15]. This might be because of VRCoNS variations in different clinical infection, geographical area, and seasonality of resistant strains in relation with the study period [51].

Enterococci showed higher and variable resistance levels to different categories of tested antibiotics. The highest resistance level among enterococci isolates was observed against ampicillin (96.2%) and lowest resistance to ciprofloxacin (53.9%). The resistance level to ampicillin in the present study was significantly higher than studies in other parts of Ethiopia which reported 54.5% to 80% of resistance [7, 23, 43] and, Nepal [37] which reported 39.5%. These variations might be because of a gradual increase in ampicillin resistance globally in the past two decades [49]. On the other hand the resistance level of erythromycin (65.4%) in the present study was comparable with reports from other parts of Ethiopia such as Gondar (64%) and Jimma (63.6%) [7, 43]. Furthermore ciprofloxacin resistance (53.9%) in the present study was in parallel with studies in Addis Ababa, Ethiopia (53.3%) and Nepal (61.5%) [8, 37]. But it was higher than a report from Jimma, Ethiopia (36.4%) and lower than Gondar, Ethiopia (70.8%) [7, 43]. These variations in AMR profile of enterococci isolates to different antibiotics might indicate different enterococcal strain distribution in different geographical areas, variability in local antibiotic prescription policy and differences in antibiotic misuse practices.

CoNS demonstrated reduced sensitivity to commonly prescribed antibiotics. This was supported by a recent CoNS meta-analysis study in Ethiopia which stated an increased antibiotic resistance level [18]. The highest resistance level among CoNS isolates were seen for ampicillin. The finding is in agreement with reports from India (72%) [40], while it was lower than reports from Jimma, Ethiopia (90.5%) [16], Iran (85%) [15], and higher than Gondar, Ethiopia (63.7%) [10]. The resistance level of CoNS for erythromycin in the present study was similar with report from Gondar, Ethiopia (51%) [10] and, Pakistan (58.3%) [41], while higher than reports from Jimma, Ethiopia (38%) [16],and Iran (40%) [15]. The resistance level of CoNS for ciprofloxacin (45.7%) was somehow in line with reports from China (52.8%) and Iran (52.5%) [15, 42]. In contrast, it was higher than studies somewhere in Ethiopia [10, 13] and Pakistan [41]. This might reflect the current increasing trend on fluoroquinolone resistance in Ethiopia mainly by CoNS [48]. On the other hand, the present study was lower than a report from India (60%) [40]. This difference might be due to the study area and setting difference.

Regarding MDR, all VRE isolates were MDR which is in line with previous studies in Ethiopia [43, 44]. The finding complements previous AMR surveillance reports which indicated VRE was a0lso resistant to multiple antibiotics [49]. However it was higher compared to reports from Nepal (31.9%) [37]. The variation might be due to differences in rational use of antibiotics, sample size and study setting. On the other hand, MDR level of CoNS was higher than reports from India (35.4%) [40], but lower than a study in Jimma, Ethiopia [16]. This study is limited due to the fact that present study didn’t detect vancomycin resistance genes.

## Conclusions

In conclusion, the prevalence of VRE in the present study was significantly higher compared with other similar studies and the pooled country prevalence and all VRE isolates were MDR. On the other hand, there were no VRCoNS isolated in the study area. But the MDR level of CoNS isolates is still significantly high. Multivariable analysis showed that educational level, previous exposure to catheterization and previous antibiotic use were significantly explained VRE infection. Hence high VRE, MDR enterococci and CoNS isolates is an alarm to health professionals for proper patient management practice since it decreases antibiotic treatment options. Thus different infection prevention measures should be implemented in the health institutes to limit development of MDR and infections due to enterococci and CoNS. Moreover large scale study is needed to further understand the vancomycin resistance level and species distribution in the health institute and community settings.

## Abbreviations

AMR: Antimicrobial resistance
AST: Antimicrobial Susceptibility Test
BSI: Blood Stream Infection
CLSI: Clinical Laboratory Standards Institute
CoNS: Coagulase-Negative Staphylococci
FHSCH: Felege-Hiwot Comprehensive Specialized Hospital
HAI: Health Care Associated Infection
IRB: Institutional Review Board
MDR: Multidrug Resistance
MIC: Minimum Inhibitory Concentration
PYR: L-pyrrolidonyl-β-arylamidase
UTI: Urinary Tract Infection
VRCoNS: Vancomycin Resistant Coagulase-Negative Staphylococci
VRE: Vancomycin Resistance Enterococci
WHO: World Health Organization
